# Supplementary material for: mTORC1 and mTORC2 differentially promote natural killer cell development
Source: eLife. 2018 May 29;7:e35619. doi: 10.7554/eLife.35619 (PMC5976438; doi:10.7554/eLife.35619)
Supplement: Supplementary file 1. [file elife-35619-supp1.docx]

**Supplementary File 1. The expression of Id2 target genes in *Rptor* cKO NK cells.**

Raptor

List gene WT WT

mean se

cKO mean

cKO

se log2FC

Adjusted P value

Significant at FDR 0.05

Id2 targets

Id3 1 0049 0.63058 0.057 -0.415 0.2624674 N

Cxcr5 1 0.102 0.32037 0.1068 -0.45 NA N

Tcf7 1 0.045 1.13758 0.1201 0.175 0.3372003 N

Tcf12 1 0.035 1.0115 0.0458 0.016 0.9314334 N

Hes1 1 0.18 1.0804 0.0707 0.097 0.790988 N

Cd3d 1 0.062 0.3802 0.0243 -1.293 7.20E-18 Y

Cd3g 1 0.026 0.41116 0.0509 -1.158 3.32E-11 Y

Socs1 1 0.052 1.48446 0.1561 0.521 0.0041257 Y

Socs3 1 0.018 1.05936 0.1034 0.079 0.6978292 N
